# The impact of maternal obesity on intrapartum outcomes in otherwise low risk women: secondary analysis of the Birthplace national prospective cohort study

**DOI:** 10.1111/1471-0528.12437

**Published:** 2013-09-11

**Authors:** J Hollowell, D Pillas, R Rowe, L Linsell, M Knight, P Brocklehurst

**Affiliations:** a National Perinatal Epidemiology Unit, University of OxfordOxford, UK; b Institute for Women's Health, University College LondonLondon, UK

**Keywords:** Adverse maternal outcomes, adverse perinatal outcomes, augmentation, caesarean section, instrumental delivery, maternal obesity, maternity unit admission criteria, parity, risk factors

## Abstract

**Objectives** To evaluate the impact of maternal BMI on intrapartum interventions and adverse outcomes that may influence choice of planned birth setting in healthy women without additional risk factors.

**Design** Prospective cohort study.

**Setting** Stratified random sample of English obstetric units.

**Sample** 17 230 women without medical or obstetric risk factors other than obesity.

**Methods** Multivariable log Poisson regression was used to evaluate the effect of BMI on risk of intrapartum interventions and adverse maternal and perinatal outcomes adjusted for maternal characteristics.

**Main outcome measures** Maternal intervention or adverse outcomes requiring obstetric care (composite of: augmentation, instrumental delivery, intrapartum caesarean section, general anaesthesia, blood transfusion, 3rd/4th degree perineal tear); neonatal unit admission or perinatal death.

**Results** In otherwise healthy women, obesity was associated with an increased risk of augmentation, intrapartum caesarean section and some adverse maternal outcomes but when interventions and outcomes requiring obstetric care were considered together, the magnitude of the increased risk was modest (adjusted RR 1.12, 95% CI 1.02–1.23, for BMI > 35 kg/m^2^ relative to low risk women of normal weight). Nulliparous low risk women of normal weight had higher absolute risks and were more likely to require obstetric intervention or care than otherwise healthy multiparous women with BMI > 35 kg/m^2^ (maternal composite outcome: 53% versus 21%). The perinatal composite outcome exhibited a similar pattern.

**Conclusions** Otherwise healthy multiparous obese women may have lower intrapartum risks than previously appreciated. BMI should be considered in conjunction with parity when assessing the potential risks associated with birth in non-obstetric unit settings.

## Introduction

Maternal obesity is a risk factor for a range of antenatal and intrapartum complications.^1^ For example, during pregnancy, obese women have an increased risk of gestational diabetes, hypertensive and thromboembolic disorders and an increased risk of miscarriage and late fetal loss; and at the time of the birth, obese women are at significantly increased risk of intrapartum caesarean section and instrumental delivery, haemorrhage, infection, longer duration of hospital stay and their infants have an increased neonatal intensive care requirement.^1^–^2^

Current clinical guidelines^3^–^4^ recommend that women with a BMI > 35 kg/m^2^ should be advised that birth in an obstetric unit would be expected to reduce the risks of maternal and fetal adverse outcomes. However, the NICE criteria for recommending birth in an obstetric unit were based on consensus rather than high quality evidence.^4^ Furthermore, the extent to which poorer intrapartum outcomes observed in obese women may be due to co-morbidities associated with obesity remains unclear.

The aim of this study was to evaluate the impact of maternal BMI on maternal interventions and adverse maternal and perinatal outcomes that may influence the choice of planned place of birth in otherwise healthy women with straightforward pregnancies of gestation of 37 weeks or more planning a vaginal birth. Specific objectives were: to describe the prevalence of medical and obstetric risk factors by maternal BMI; to evaluate the association between maternal BMI and intrapartum interventions and adverse maternal and perinatal outcomes both overall and in otherwise healthy women without known medical and obstetric risk factors immediately prior to the onset of labour; to explore whether the effects of BMI differ by parity; and to explore the relationships between maternal BMI and maternal and perinatal outcomes in planned home and midwifery unit births.

## Methods

### Setting and participants

The study used data from the Birthplace in England national prospective cohort study which was designed to compare perinatal and maternal outcomes and interventions by planned place of birth at the start of care in labour.

The cohort study methods and data are described in full elsewhere.^5^–^6^ Briefly, the Birthplace cohort includes a total of 79 774 births between April 2008 and April 2010, including 32 257 planned obstetric unit births from a stratified random sample of 36 obstetric units, 11 666 planned births in 53 freestanding midwifery units, 17 582 planned births in 43 alongside midwifery units and 18 269 planned home births from 142 NHS trusts across England. Births were eligible for inclusion if the woman was planning a vaginal birth and received some labour care from an NHS midwife in her planned birth setting. Women who had an elective caesarean section or caesarean section before the onset of labour, presented in preterm labour (<37 weeks' gestation), had a multiple pregnancy, or who were ‘unbooked’ (received no antenatal care) were excluded. Stillbirths occurring before the start of care in labour were excluded. The study achieved a high response rate with a low level of missing data.^5^–^6^

Research ethics committee approval for the Birthplace study was obtained from the Berkshire Research Ethics Committee and did not require consent to be sought from participants as no personally identifiable data were collected.

Women in the obstetric unit group were used as the main analysis population for this study since women with a BMI > 35 kg/m^2^ are usually advised to plan birth in an obstetric unit and hence are substantially under-represented in other settings.

### Outcomes

We focused on outcome measures that reflected interventions and adverse outcomes that indicated a need for obstetric and/or neonatal care. For the woman, we considered the following interventions and adverse maternal outcomes requiring obstetric care, both separately and as a combined composite outcome measure: augmentation with syntocinon, instrumental delivery, intrapartum caesarean section, general anaesthesia, maternal blood transfusion, third/fourth degree perineal tear, maternal admission for higher level care.

For the baby, we considered a single composite outcome measure encompassing admission to a neonatal unit within 48 hours of birth, stillbirth after the start of care in labour or early neonatal death. We chose an outcome focusing on neonatal unit admission rather than the original Birthplace primary outcome measure (a composite measure of intrapartum related adverse perinatal outcome encompassing intrapartum stillbirth, early neonatal death, neonatal encephalopathy, meconium aspiration syndrome, brachial plexus injury and fractured humerus or clavicle) for two reasons. First, the event rate for intrapartum related adverse perinatal outcomes (4.3 adverse perinatal outcomes per 1000 births in ‘low risk’ women) was too low for us to have adequate statistical power to detect less than a tripling of risk in the group of women with a BMI > 35 kg/m^2^. Second, because our focus was on outcomes that might influence planned place of birth, we required a measure that captured need for neonatal care, and there are reasons why a baby may require ready access to neonatal care that are not captured by the original Birthplace measure.

### Data and definitions

As described elsewhere,^6^ maternal characteristics, including BMI in pregnancy, and medical or obstetric risk factors known prior to the onset of labour were extracted from the woman's medical records by the midwife attending the birth. Complicating conditions identified by the midwife at the start of care in labour (for example prolonged rupture of membranes), intrapartum interventions and adverse maternal and perinatal outcomes were recorded prospectively by the attending midwife using a data collection form started during labour and completed on or after the fifth postnatal day.

Women were classified into the following BMI groups using standard cut-offs^6^: underweight (BMI < 18.5 kg/m^2^), normal (BMI 18.5–24.9 kg/m^2^), overweight (BMI 25–29.9 kg/m^2^), obese (BMI 30–35 kg/m^2^), very obese (BMI > 35 kg/m^2^). Where the midwife explicitly reported that BMI was not recorded in the woman's notes, BMI was classified as ‘not recorded’ rather than ‘missing’ since it was assumed that these would not be missing at random.

Women were classified as ‘healthy women without additional risk factors’ if, before the onset of labour, they were not known to have any of the medical or obstetric risk factors listed in the NICE intrapartum care guideline, other than BMI > 35 kg/m^2^. These ‘risk factors’ (see appendix Tables S1 and S2 for a list of conditions) are considered to increase risk for the woman or baby, and care in an obstetric unit would be expected to reduce this risk.^7^

### Statistical analysis

Women in the obstetric unit group for whom BMI was recorded formed the analysis population for this study. This population included both ‘low risk’ women and women with known pre-existing risk factors. The main analyses reported here were conducted in the restricted sample of healthy women without additional risk factors, i.e. with no risk factors other than BMI > 35 kg/m^2^. Analyses were additionally conducted in women in the obstetric unit group irrespective of the presence or absence of other risk factors (the ‘all risks’ sample).

Robust variance estimation was used to allow for the clustered nature of the data. As described elsewhere,^6^ probability weights were used to account for differences in the probability of a woman being selected for inclusion in the study arising from differences in each unit or trust's period of participation and the stratum-specific probabilities of selection of obstetric units.

We aimed to use binomial regression to calculate relative risks and 95% confidence intervals and to use log Poisson regression if the binomial regression failed to converge.^8^ In practice, the binomial models consistently failed to converge once adjustment factors were added so log Poisson regression was used throughout. Analyses were adjusted for maternal age, ethnic group, understanding of English, marital or partner status, index of multiple deprivation (IMD) score, parity and gestational age at birth using the categories shown in supplementary Table S3 (See appendix). For each outcome, we report the number of events, the number of births, the weighted incidence and the unadjusted and adjusted relative risks. For completeness, the unadjusted relative risks restricted to births included in the adjusted analysis are also reported in the supplementary tables. Women with a normal maternal BMI were the reference category in all analyses.

To ascertain whether the associations between BMI and the two composite outcome measures differed for nulliparous and multiparous women, a Wald test for statistical interaction was performed, with a 5% significance level being accepted as evidence of an interaction.

Because the primary cohort study analyses^6^ showed that maternal intervention rates tend to be significantly lower in births planned in non-obstetric unit settings, we repeated the main analyses (maternal and perinatal composites only) for the planned home, Freestanding Midwifery Unit and Alongside Midwifery Unit births, restricted to healthy women without additional risk factors. For this analysis, the very obese group was restricted to women with a BMI ≤ 40 kg/m^2^ because previous analysis^5^ showed that few women with a BMI above this value plan birth in a non-obstetric unit setting.

In order to explore the relationships between BMI as a continuous variable and selected outcomes, we plotted the unadjusted proportion of births in which the outcome occurred against BMI, using the locally weighted scatterplot smoothing (LOWESS) technique, with a bandwidth of 0.5.^9^ stata version 11.2 was used for all analyses.^10^

## Results

In total there were 26 904 women with BMI recorded in the obstetric unit group (‘all risks’ sample). BMI was not recorded in 17.2% of records and missing in 0.3% of records.

In the ‘all risks’ sample, 36% of women (*n* = 9674) had pre-existing risk factors other than BMI > 35 kg/m^2^. The proportion of women with pre-existing medical risk factors increased with BMI category from 8% in underweight women to 14% in very obese women, and the proportion of women with pre-existing obstetric or fetal risk factors (other than BMI > 35 kg/m^2^) increased from 24% in underweight women to 41% in very obese women. Amongst very obese women the proportion of women with at least one pre-existing risk factor other than BMI > 35 kg/m^2^ was 48%. The prevalence of individual risk factors is tabulated in Table S4.

The characteristics of the main study sample of healthy women without additional risk factors (*n* = 17 230) varied across BMI categories (Table 1). Compared with women of normal weight, women who were overweight, obese or very obese were more likely to be white, have a fluent understanding of English, live in a more socioeconomically deprived area, and be multiparous. The number of previous pregnancies tended to increase with increasing BMI. There were some differences in gestational age by BMI category but no clear trend was evident. Birth weight and the proportion of babies weighing over 4000 g and over 4500 g increased with increasing BMI. Underweight women were more likely to be younger, non-white, not have a fluent understanding of English, be single/unsupported by their partner, live in a more socioeconomically deprived area, and be nulliparous.

**Table 1:** Characteristics of healthy women without additional risk factors by maternal BMI category

	Underweight *n* = 577		Normal weight *n* = 8936		Overweight *n* = 4778		Obese *n* = 1955		Very obese *n* = 984	
	*n*	%	*n*	%	*n*	%	*n*	%	*n*	%
**Maternal age (years)**										
Mean [SD]	25.4	[5.6]	28.0	[6.0]	28.8	[5.9]	28.2	[5.8]	28.1	[5.9]
Under 20	96	16.6	763	8.5	270	5.7	120	6.1	53	5.4
20–24	182	31.5	1934	21.6	934	19.5	463	23.7	266	27.0
25–29	153	26.5	2575	28.8	1430	29.9	564	28.8	287	29.2
30–34	106	18.4	2300	25.7	1234	25.8	500	25.6	212	21.5
35–39	39	6.8	1134	12.7	754	15.8	255	13.0	126	12.8
40+	1	0.2	223	2.5	153	3.2	50	2.6	37	3.8
Missing	0	0.0	7	0.1	3	0.1	3	0.2	3	0.3
**Ethnic group**										
White	451	78.2	7298	81.7	3894	81.5	1630	83.4	863	87.7
Indian	21	3.6	213	2.4	94	2.0	26	1.3	7	0.7
Pakistani	23	4.0	294	3.3	182	3.8	65	3.3	20	2.0
Bangladeshi	20	3.5	150	1.7	82	1.7	18	0.9	3	0.3
Black Caribbean	9	1.6	107	1.2	84	1.8	24	1.2	22	2.2
Black African	7	1.2	239	2.7	187	3.9	93	4.8	40	4.1
Mixed	10	1.7	134	1.5	74	1.5	30	1.5	15	1.5
Other	35	6.1	488	5.5	180	3.8	64	3.3	13	1.3
Missing	1	0.2	13	0.1	1	0.0	5	0.3	1	0.1
**Understanding of English**										
Fluent	495	85.8	8148	91.2	4387	91.8	1839	94.1	958	97.4
Some	56	9.7	567	6.3	253	5.3	80	4.1	19	1.9
None	21	3.6	180	2.0	112	2.3	25	1.3	6	0.6
Missing	5	0.9	41	0.5	26	0.5	11	0.6	1	0.1
**Marital/partner status**										
Married/Living together	480	83.2	7708	86.3	4208	88.1	1691	86.5	826	83.9
Single/Unsupported by partner	91	15.8	1073	12.0	490	10.3	226	11.6	142	14.4
Missing	6	1.0	155	1.7	80	1.7	38	1.9	16	1.6
**Index of Multiple Deprivation quintiles**										
1st Least deprived	57	9.9	1467	16.4	719	15.0	247	12.6	124	12.6
2nd	85	14.7	1725	19.3	834	17.5	317	16.2	148	15.0
3rd	102	17.7	1696	19.0	913	19.1	337	17.2	173	17.6
4th	145	25.1	1811	20.3	976	20.4	454	23.2	229	23.3
5th Most deprived	184	31.9	2182	24.4	1293	27.1	588	30.1	297	30.2
Missing	4	0.7	55	0.6	43	0.9	12	0.6	13	1.3
**Previous pregnancies ≥24 weeks**										
0 Nulliparous	344	59.6	5005	56.0	2420	50.6	945	48.3	418	42.5
1 previous	168	29.1	2602	29.1	1431	29.9	590	30.2	297	30.2
2 previous	43	7.5	834	9.3	545	11.4	242	12.4	164	16.7
3+ previous	22	3.8	484	5.4	371	7.8	174	8.9	104	10.6
Missing	0	0.0	11	0.1	11	0.2	4	0.2	1	0.1
**Gestation (completed weeks)**										
Mean [SD]	39.5	[1.2]	39.7	[1.1]	39.8	[1.1]	39.9	[1.1]	39.9	[1.1]
37	37	6.4	351	3.9	160	3.3	51	2.6	23	2.3
38	79	13.7	860	9.6	489	10.2	149	7.6	88	8.9
39	167	28.9	2138	23.9	1046	21.9	388	19.8	219	22.3
40	179	31.0	3219	36.0	1660	34.7	699	35.8	323	32.8
41	98	17.0	2139	23.9	1268	26.5	599	30.6	297	30.2
42–44	16	2.8	210	2.4	144	3.0	62	3.2	33	3.4
Missing	1	0.2	19	0.2	11	0.2	7	0.4	1	0.1
**Birthweight (grams)**										
Mean [SD]	3202	[421.1]	3418	[447.1]	3505	[467.4]	3573	[478.4]	3594	[467.5]
>2500	24	4.2	128	1.4	59	1.2	18	0.9	5	0.5
2500–2999	153	26.5	1394	15.6	566	11.8	204	10.4	81	8.2
3000–3499	267	46.3	3690	41.3	1792	37.5	642	32.8	339	34.5
3500–3999	110	19.1	2819	31.5	1667	34.9	722	36.9	363	36.9
4000–4499	17	2.9	784	8.8	582	12.2	310	15.9	163	16.6
≥4500	5	0.9	106	1.2	105	2.2	53	2.7	32	3.3
Missing	1	0.2	15	0.2	7	0.1	6	0.3	1	0.1

In healthy women without additional risk factors, the proportion of women found to have a complicating condition at the start of care in labour increased from 18.2% in women of normal weight to 23.4% in very obese women (Table 2). Prolonged rupture of membranes and meconium staining were the two most commonly noted complications.

**Table 2:** Complicating conditions identified at the start of care in labour by maternal BMI category in healthy women without additional risk factors

	Underweight *n* = 575		Normal weight *n* = 8890		Overweight *n* = 4757		Obese *n* = 1947		Very obese *n* = 982	
	*n*	%	*n*	%	*n*	%	*n*	%	*n*	%
Prolonged rupture of membranes >18 hours	36	6.26	645	7.26	387	8.14	161	8.27	72	7.33
Meconium-stained liquor	34	5.91	514	5.78	316	6.64	149	7.65	89	9.06
Proteinuria 1+ or more	3	0.52	148	1.66	88	1.85	52	2.67	40	4.07
Hypertension*	9	1.57	188	2.11	142	2.99	94	4.83	38	3.87
Abnormal vaginal bleeding	13	2.26	110	1.24	69	1.45	17	0.87	8	0.81
Non-cephalic presentation	3	0.52	48	0.54	29	0.61	5	0.26	3	0.31
Abnormal fetal heart rate	10	1.74	162	1.82	106	2.23	54	2.77	20	2.04
Other complications	0	–	21	0.24	17	0.36	7	0.36	2	0.20
Any complicating condition	97	16.87	1619	18.21	999	21.00	460	23.63	230	23.42

* Either diastolic blood pressure of ≥90 mmHg on more than one occasion 20 minutes apart or ≥100 mmHg on one occasion; or systolic blood pressure ≥160 mmHg on at least one occasion.

### Perinatal outcomes

The incidence of admission to a neonatal unit or intrapartum stillbirth/early neonatal death increased with BMI from 2.4% in underweight women to 4.7% in very obese women (Table 3). After adjustment for maternal characteristics, the risk to the baby was significantly raised only in very obese women (RR 1.90, 95% CI 1.41–2.56). The BMI gradient did not differ by parity (test for interaction *P* = 0.84) but absolute risks to the baby were higher in nulliparous women: for example, the rate of neonatal admission/perinatal death was 7.1% (95% CI 4.3–9.9) in otherwise healthy very obese nulliparous women compared with 2.9% (95% CI 1.5–4.4) in otherwise healthy very obese multiparous women (Table 3).

**Table 3:** Admission to a neonatal unit or intrapartum stillbirth/early neonatal death by maternal BMI category in healthy women without additional risk factors overall and by parity

	Events	Births	Weighted*		Unadjusted*		Adjusted*,**	
	*n*	*n*	%	(95% CI)	RR	(95% CI)	RR	(95% CI)
**All**								
Underweight	14	576	2.4	(1.4–3.4)	0.84	(0.50–1.39)	0.82	(0.48–1.39)
Normal weight	249	8881	2.8	(2.2–3.5)	1	–	1	–
Overweight	123	4750	2.7	(2.0–3.5)	0.97	(0.76–1.23)	0.96	(0.75–1.23)
Obese	58	1946	3.0	(2.1–4.0)	1.06	(0.76–1.47)	1.17	(0.85–1.62)
Very obese	43	981	4.7	(3.2–6.2)	1.64	(1.22–2.21)	1.90	(1.41–2.56)
Total	487	17 134	2.9	(2.4–3.5)				
Nulliparous***								
Underweight	9	344	2.6	(0.9–4.3)	0.70	(0.35–1.40)	0.72	(0.36–1.46)
Normal weight	180	4979	3.7	(2.8–4.6)	1	–	1	–
Overweight	76	2406	3.3	(2.1–4.6)	0.90	(0.62–1.30)	0.88	(0.62–1.24)
Obese	39	938	4.1	(2.5–5.8)	1.11	(0.75–1.65)	1.18	(0.80–1.74)
Very obese	28	417	7.1	(4.3–9.9)	1.90	(1.24–2.91)	2.00	(1.31–3.05)
Total	332	9084	3.8	(3.0–4.6)				
Multiparous***								
Underweight	5	232	2.0	(0.4–3.7)	1.20	(0.48–2.98)	1.13	(0.40–3.19)
Normal weight	68	3891	1.7	(1.2–2.2)	1	–	1	–
Overweight	46	2333	2.1	(1.5–2.7)	1.23	(0.92–1.63)	1.19	(0.88–1.61)
Obese	19	1005	2.0	(1.0–2.9)	1.16	(0.65–2.07)	1.26	(0.69–2.28)
Very obese	15	563	2.9	(1.5–4.3)	1.69	(1.13–2.53)	1.83	(1.22–2.75)
Total	153	8024	1.9	(1.5–2.4)				

* Weighted to reflect each unit's duration of participation and the sampling of obstetric units.

** Adjusted for maternal age, ethnic group, understanding of English, marital/partner status, index of multiple deprivation score quintile, previous pregnancies ≥24 weeks, and gestation (completed weeks).

*** Wald test for interaction with parity: *P *= 0.84.

### Maternal interventions and adverse outcomes

Analysis of individual interventions and adverse maternal outcomes in healthy women without additional risk factors showed that the risk of intrapartum caesarean section, augmentation and general anaesthesia all increased with BMI, with overweight, obese and very obese woman all at significantly increased risk of intrapartum caesarean section and augmentation (Table 4). The reverse was observed for instrumental deliveries, where overweight and obese women were at reduced risk of an instrumental delivery (forceps or ventouse). There were no consistent, statistically significant associations between BMI and maternal blood transfusion, third/fourth degree tear or maternal admission for higher level care.

**Table 4:** Obstetric interventions and adverse maternal outcomes by maternal BMI category in healthy women without additional risk factors

	Events	Births	Weighted*		Unadjusted*		Adjusted*,**	
	*n*	*n*	%	(95% CI)	RR	(95% CI)	RR	(95% CI)
**Instrumental delivery**								
Underweight	79	577	14.0	(11.0–17.0)	0.86	(0.72–1.02)	0.95	(0.79–1.13)
Normal weight	1397	8928	16.3	(13.9–18.7)	1	–	1	–
Overweight	635	4774	13.5	(11.8–15.2)	0.83	(0.76–0.91)	0.87	(0.80–0.95)
Obese	249	1951	12.8	(10.8–14.9)	0.79	(0.67–0.93)	0.86	(0.74–1.00)
Very obese	84	983	9.4	(7.0–11.7)	0.57	(0.46–0.71)	0.70	(0.57–0.86)
Total	2444	17 213	14.7	(12.7–16.6)				
**Intrapartum caesarean section**								
Underweight	39	577	6.9	(4.5–9.4)	0.72	(0.54–0.97)	0.83	(0.61–1.13)
Normal weight	846	8928	9.6	(8.3–10.9)	1	–	1	–
Overweight	588	4774	12.5	(11.1–13.8)	1.30	(1.16–1.46)	1.34	(1.20–1.50)
Obese	260	1951	13.6	(11.7–15.6)	1.42	(1.24–1.64)	1.52	(1.30–1.79)
Very obese	135	983	13.6	(11.2–16.0)	1.42	(1.16–1.74)	1.69	(1.35–2.12)
Total	1868	17 213	11.0	(9.8–12.1)				
**Augmentation**								
Underweight	102	571	17.4	(13.7–21.1)	0.75	(0.61–0.93)	0.80	(0.66–0.98)
Normal weight	2024	8823	23.2	(21.2–25.1)	1	–	1	–
Overweight	1152	4753	24.6	(22.1–27.1)	1.06	(1.00–1.12)	1.10	(1.03–1.16)
Obese	519	1930	27.3	(23.7–30.9)	1.18	(1.08–1.29)	1.26	(1.16–1.37)
Very obese	266	972	27.2	(22.9–31.5)	1.18	(1.03–1.35)	1.35	(1.20–1.53)
Total	4063	17 031	24.1	(21.9–26.2)				
**General anaesthesia**								
Underweight	8	559	1.4	(0.3–2.6)	1.09	(0.51–2.34)	1.16	(0.53–2.54)
Normal weight	117	8805	1.3	(1.0–1.6)	1	–	1	–
Overweight	80	4707	1.7	(1.2–2.1)	1.25	(0.94–1.68)	1.25	(0.93–1.69)
Obese	31	1920	1.6	(1.0–2.2)	1.19	(0.79–1.81)	1.19	(0.78–1.81)
Very obese	20	971	2.1	(1.0–3.2)	1.62	(0.96–2.73)	1.79	(1.04–3.07)
Total	256	16 972	1.5	(1.2–1.8)				
**Maternal blood transfusion**								
Underweight	6	574	1.2	(0.4–2.1)	0.94	(0.44–2.02)	1.03	(0.48–2.21)
Normal weight	112	8881	1.3	(1.0–1.7)	1	–	1	–
Overweight	61	4735	1.2	(0.9–1.6)	0.91	(0.61–1.35)	0.96	(0.62–1.48)
Obese	25	1945	1.2	(0.7–1.8)	0.94	(0.63–1.40)	1.00	(0.65–1.53)
Very obese	9	984	0.9	(0.4–1.4)	0.66	(0.34–1.28)	0.77	(0.40–1.50)
Total	213	17 119	1.3	(1.0–1.5)				
**Third/fourth-degree tear**								
Underweight	20	571	3.6	(2.3–5.0)	1.10	(0.75–1.60)	1.15	(0.78–1.68)
Normal weight	301	8903	3.3	(2.8–3.9)	1	–	1	–
Overweight	139	4759	2.9	(2.4–3.5)	0.88	(0.74–1.04)	0.93	(0.78–1.10)
Obese	48	1947	2.4	(1.7–3.1)	0.73	(0.53–1.02)	0.82	(0.60–1.13)
Very obese	24	983	2.4	(1.3–3.7)	0.75	(0.47–1.20)	0.86	(0.56–1.33)
Total	532	17 163	3.1	(2.6–3.5)				
**Maternal admission for higher level care**								
Underweight	5	577	1.0	(0.1–1.9)	1.46	(0.60–3.58)	1.63	(0.72–3.69)
Normal weight	57	8936	0.7	(0.3–1.0)	1	–	1	–
Overweight	28	4778	0.6	(0.2–0.9)	0.80	(0.42–1.50)	0.78	(0.41–1.49)
Obese	11	1955	0.7	(0.2–1.1)	0.96	(0.56–1.64)	0.88	(0.50–1.54)
Very obese	5	984	0.5	(0.1–0.9)	0.68	(0.26–1.74)	0.71	(0.25–2.03)
Total	106	17 230	0.6	(0.4–0.9)				

* Weighted to reflect each unit's duration of participation and the sampling of obstetric units.

** Adjusted for maternal age, ethnic group, understanding of English, marital/partner status, index of multiple deprivation score quintile, previous pregnancies ≥24 weeks, and gestation (completed weeks).

Overall, 37.7% of healthy women of normal weight experienced an obstetric intervention or adverse maternal outcome: the adjusted risk was significantly increased in overweight, obese and very obese women but the increase in risk was modest (6–12%) (Table 5).

**Table 5:** Obstetric interventions and adverse maternal outcomes combined by maternal BMI category and parity in healthy women without additional risk factors

	Events	Births	Weighted*		Unadjusted*		Unadjusted*^,^**	
	*n*	*n*	%	(95% CI)	RR	(95% CI)	RR	(95% CI)
**All**								
Underweight	182	558	32.8	(28.9–36.7)	0.88	(0.78–0.99)	0.94	(0.84–1.04)
Normal weight	3192	8648	37.5	(35.1–39.8)	1	–	1	–
Overweight	1747	4621	38.2	(35.8–40.6)	1.02	(0.97–1.07)	1.06	(1.01–1.11)
Obese	748	1885	40.1	(36.7–43.4)	1.07	(0.99–1.15)	1.14	(1.08–1.20)
Very obese	342	960	36.4	(31.9–40.8)	0.97	(0.87–1.08)	1.12	(1.02–1.23)
Total	6211	16 672	37.7	(35.6–39.9)				
Nulliparous***								
Underweight	150	330	45.6	(39.5–51.7)	0.86	(0.76–0.98)	0.94	(0.82–1.09)
Normal weight	2524	4833	52.9	(50.3–55.4)	1	–	1	–
Overweight	1277	2321	55.7	(52.4–59.0)	1.05	(1.01–1.10)	1.04	(0.99–1.08)
Obese	535	907	60.2	(55.9–64.4)	1.14	(1.07–1.21)	1.12	(1.05–1.18)
Very obese	225	404	57.1	(52.2–62.0)	1.08	(0.98–1.19)	1.08	(0.99–1.18)
Total	4711	8795	54.3	(51.8–56.8)				
Multiparous***								
Underweight	32	228	14.6	(8.1–21.1)	0.83	(0.55–1.24)	0.87	(0.57–1.31)
Normal weight	666	3809	17.7	(15.7–19.7)	1	–	1	–
Overweight	465	2290	20.2	(17.7–22.7)	1.14	(1.00–1.30)	1.16	(1.02–1.32)
Obese	212	975	21.3	(17.6–25.0)	1.20	(1.01–1.44)	1.22	(1.05–1.42)
Very obese	117	555	21.0	(15.1–26.9)	1.19	(0.90–1.57)	1.24	(0.97–1.59)
Total	1492	7857	19.0	(17.1–21.0)				

* Weighted to reflect each unit's duration of participation and the sampling of obstetric units.

** Adjusted for maternal age, ethnic group, understanding of English, marital/partner status, index of multiple deprivation score quintile, previous pregnancies ≥24 weeks, and gestation (completed weeks).

*** Wald test for interaction with parity: *P* = 0.24.

In healthy women without additional risk factors, the relationship between BMI and the composite maternal outcome (obstetric interventions and adverse maternal outcomes combined) did not differ significantly by parity (Wald test for interaction *P* = 0.24). In healthy women without additional risk factors, risks were such that a nulliparous woman of normal weight had a higher absolute risk of an obstetric intervention or adverse maternal outcome than a multiparous very obese woman: 53% of healthy nulliparous women of normal weight experienced one of these outcomes versus 21% of otherwise healthy obese or very obese multiparous women (Table 5). Adjustment for maternal characteristics confirmed that the risk of an obstetric intervention or adverse maternal outcome was significantly higher in nulliparous women of normal weight compared to multiparous women in any of the BMI categories considered (e.g. relative risk 0.28, 95% CI 0.20–0.38 for multiparous very obese women versus nulliparous women of normal weight).

**Figure 1:**
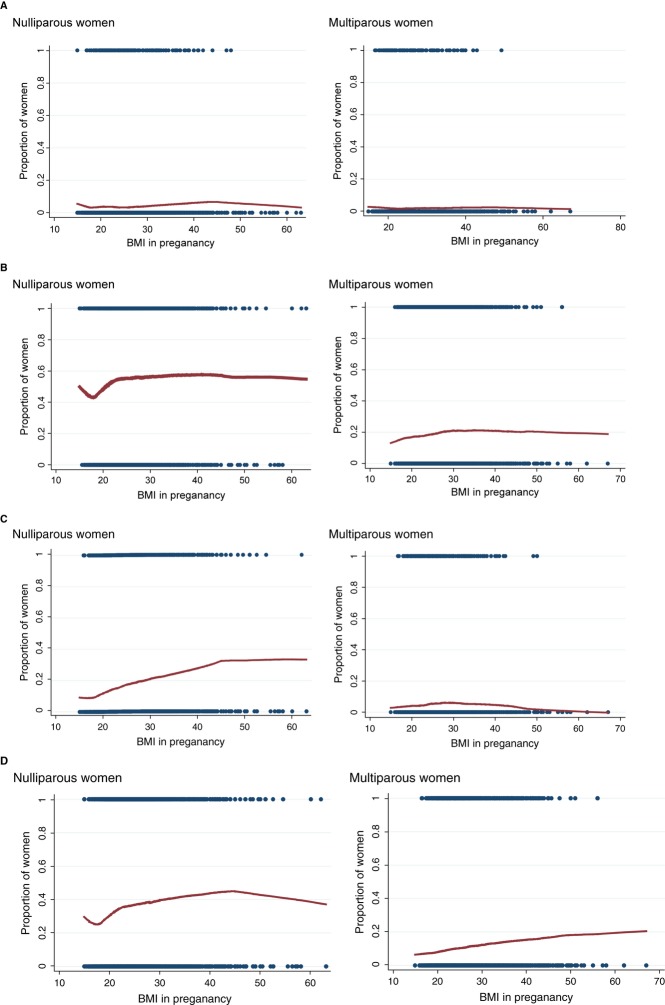
LOWESS plots of perinatal and maternal outcomes by BMI and parity in healthy women without additional risk factors. (A) Admission to a neonatal unit or intrapartum stillbirth/early neonatal death (composite perinatal outcome). (B) Obstetric interventions and adverse maternal outcomes combined (composite maternal outcome). (C) Intrapartum caesarean section. (D) Syntocinon augmentation.For each BMI value on the x-axis, the red line on the LOWESS plot^9^ shows the unadjusted proportion of births where the outcome occurred. Peaks and troughs caused by small numbers of observations at a particular BMI value are smoothed by using regression methods to take account of values close to the BMI of interest. Individual observations (which take the value one if the event has occurred, zero otherwise) are plotted as black dots at zero and one. The density of the line at zero and one indicates the density of observations at a particular BMI value: the lines become less dense at higher BMI values where observations are sparse.

LOWESS plots for the maternal composite outcome showed that for healthy nulliparous and multiparous women of normal weight or above, the probability of an obstetric intervention or adverse maternal outcome appeared to plateau at around a BMI of 30 (Figure 1). Otherwise healthy nulliparous women who were very underweight also appeared to be at increased risk of an obstetric intervention or adverse maternal outcome compared to women who were borderline underweight. Different patterns were observed for individual interventions. The probability of intrapartum caesarean section, for example, tended to plateau or decrease above a BMI of around 30 in otherwise healthy multiparous women but did not appear to plateau with BMI in otherwise healthy nulliparous women (Figure 1).

### Outcomes in planned births in non-obstetric unit settings

In healthy women without additional risk factors, no consistent relationship with BMI was observed for either the perinatal or maternal composite outcome measures in other planned birth settings (Tables S9–S10), although lack of a consistent pattern could be due to the much smaller numbers of obese women planning births in these settings. There was a significant association between being very obese and the perinatal composite outcome for planned Freestanding Midwifery Unit births.

### Outcomes in the ‘all risk’ sample

Broadly similar patterns were observed when women with additional risk factors were included in the analysis, although in general the BMI gradient for most outcomes was steeper in the ‘all risks’ sample (Tables S5–S6, and S11–S12).

## Discussion

### Main findings

In women without additional risk factors we found that obesity was associated with an increased risk of some maternal interventions, including augmentation with syntocinon and intrapartum caesarean section, and an increased risk of some adverse maternal outcomes. Obese women had a reduced risk of instrumental delivery. Taking all interventions and adverse maternal outcomes requiring obstetric care together, overweight, obese and very obese women were at significantly increased risk but the increase was fairly modest. In otherwise healthy women, the risk of any of these outcomes increased by <15% relative to women of normal weight after adjustment for maternal characteristics.

The risks associated with nulliparity were such that a ‘low risk’ nulliparous women of normal weight had a higher risk of an obstetric intervention or adverse maternal outcome requiring obstetric care than an otherwise ‘low risk’ obese or very obese multiparous woman (52.9% versus 21.0%). Absolute risks to the baby (admission to a neonatal unit or intrapartum stillbirth/early neonatal death) followed a similar pattern with higher event rates in nulliparous women of normal weight compared with those in very obese otherwise healthy multiparous women (3.7% versus 2.9%).

### Strengths and limitations

Strengths of this study are that it is based on a large sample of births drawn from a nationally representative sample of obstetric units in England, with high quality data on interventions and other outcomes collected prospectively by the attending midwives. We lacked the statistical power to investigate the incidence of uncommon intrapartum related adverse perinatal outcomes, such as neonatal encephalopathy and brachial plexus injury, which have a low incidence in ‘low risk’ women (2.2 and 0.4 per 1000 births respectively in the Birthplace cohort^6^) Instead, we used a composite measure combining admission to a neonatal unit and intrapartum stillbirth/early neonatal death to assess adverse perinatal outcomes. Rates of admission to a neonatal unit may be affected by factors other than severe morbidity, but admission to higher level neonatal care is a relevant outcome when considering whether it is appropriate for a woman to plan birth in a setting where neonatal care is not immediately available.

Additional strengths of the study are that we have been able to assess the independent effect of overweight and obesity by assessing risks in otherwise healthy women without additional risk factors for adverse neonatal or maternal outcomes, and have also been able to assess the effect of parity on risks associated with BMI. Other studies have evaluated risks in ‘lower risk’ cohorts, but we are not aware of other studies that have excluded the effect of potential risk factors in such a comprehensive way, or that have directly compared absolute risks associated with overweight and obesity in nulliparous and multiparous women.

A limitation is that data on BMI in pregnancy were extracted from the woman's medical record and we lack information on when this was recorded (typically booking). Additionally, although there appears to be no reason why this should have biased our findings, 17.2% of eligible women had to be excluded from the analysis because BMI had not been recorded in the medical record, despite clinical guidelines.^11^

Our cohort included only women planning a vaginal birth and outcomes in this group are likely to be affected by background caesarean section rates. Hence the findings may not be generalisable to other settings with different caesarean section rates or practice patterns.

### Other evidence and clinical implications

We found that the incidence of common obstetric interventions was substantially lower in overweight and obese women when women with additional risk factors such as diabetes and hypertension were excluded, suggesting that the higher incidence of these outcomes observed in overweight and obese women^2^–^12^ was partly attributable to the medical and obstetric risk factors associated with a high BMI.

We lacked data on the nature of clinical complications during labour but the frequency of augmentation of labour suggests that failure to progress may have been the presenting problem in the majority (up to three quarters) of the cases where healthy obese and very obese women experienced outcomes that required obstetric care. This is consistent with a body of evidence indicating that obese women have less effective uterine contractility and longer labours,^13^–^14^ are more likely to experience failure to progress/labour arrest^2^–^15^ and have an increased risk of non-elective section for labour arrest disorders^14^,^16^ or ‘failure to progress/cephalopelvic disproportion’.^18^

Labour disorders have been found to be significantly less common in multiparous obese women compared with nulliparous women.^14^–^20^ This is consistent with the lower rates of augmentation observed in multiparous women in our study.

We do not know what proportion of women in our study had complications that required more urgent obstetric attention. Studies including women with additional medical and obstetric risk have reported an association between BMI and caesarean section for fetal distress in nulliparous women,^16^–^21^ but other studies have not found an increased risk.^13^–^22^

Evidence from studies that have evaluated outcomes in various ‘lower risk’ cohorts suggests that BMI does have an independent effect on caesarean section,^23^–^27^ and on emergency caesarean section in nulliparous women.^28^–^29^ Our findings therefore add to the body of evidence that overweight and obesity are independent risk factors for caesarean section in otherwise healthy overweight, obese and very obese women.^23^–^27^

Obesity has been shown to have an independent effect on macrosomia/large for gestational age (LGA)^26^–^30^ which, in turn, is strongly associated with shoulder dystocia.^31^–^32^ However, although UK clinical guidelines state that women with obesity are at significantly increased risk of shoulder dystocia,^3^ the evidence does not clearly show that obesity is an independent risk factor. A meta-analysis of studies identified by systematic searches did not show a significantly increased risk of shoulder dystocia in obese women (odds ratio 1.04, 95% CI 0.97–1.16).^2^ The risk of neonatal injury associated with shoulder dystocia may, however, be affected by maternal obesity.^33^

Women who are obese have been found to be at increased risk of postpartum haemorrhage,^2^ probably predominantly related to poorer uterine contractility/uterine atony^17^,^19^ and in part to the increased incidence of obstetric interventions in obese women.^34^–^35^ In our study we did not observe an increase in the incidence of maternal blood transfusion with BMI possibly reflecting the high level of active management of the third stage of labour (around 95%^6^) in the obstetric unit sample.

Finally, although many studies exploring the impact of obesity note that nulliparity is a risk factor for a range of adverse intrapartum outcomes, few studies have explicitly addressed the risks of intrapartum complications associated with BMI and parity in combination. One small study reported the incidence of interventions and adverse outcomes by BMI and parity in women with uncomplicated pregnancies and found patterns consistent with our findings; and a study designed to develop a ‘predictor’ of peripartum complications associated with BMI similarly found that some obese multiparous women (particularly younger obese women) were at lower risk of intrapartum complications than some non-obese nulliparous women.^36^

## Conclusions and implications for policy and practice

Our results suggest that obese multiparous women who do not have additional risk factors, such as diabetes or previous caesarean section, may have lower obstetric risks than previously appreciated. Further research may be required to determine whether adverse perinatal outcomes associated with shoulder dystocia are also reduced in this ‘otherwise healthy’ group. If so, it may be reasonable to review the BMI criteria for planned birth in non-obstetric unit settings, particularly Alongside Midwifery Units where obstetric and neonatal care is available on site if needed. More generally, parity should be taken into account when assessing the potential risks associated with birth in non-obstetric unit settings.

### Disclosure of interests

No conflicting interests to declare.

### Contribution to authorship

JH and PB conceived and developed the study outline; JH and DP developed the protocol and analysis plan; DP conducted the analysis; LL provided statistical advice; JH drafted the manuscript with input from DP and RR; JH, DP, RR, LL, MK and PB were involved in interpretation of data, review and revision of the draft manuscript and approval of the final version.

### Details of ethics approval

Research ethics committee approval for the Birthplace study was obtained from the Berkshire Research Ethics Committee (MREC ref 07/H0505/151).

### Funding

This paper reports on an independent study which is funded/part-funded by the Policy Research Programme in the Department of Health. The views expressed are not necessarily those of the Department. Birthplace combines the Evaluation of Maternity Units in England study funded in 2006 by the National Institute for Health Research Service Delivery and Organisation (NIHR SDO) programme, and the Birth at Home in England study funded in 2007 by the Department of Health Policy Research Programme (DH PRP). From January 2012, the NIHR SDO programme merged with the NIHR Health Services Research programme to establish the new NIHR health Services and Delivery Research (NIHR HS&DR) programme. The views and opinions expressed by the authors do not necessarily reflect those of the HS&DR Programme, NIHR, NHS, DH PRP or the Department of Health.

## Supporting Information

**Table S1**. Medical ‘risk factors’ (from NICE intrapartum care guideline).^4^ **Table S2**. Obstetric history ‘risk factors’ (from NICE intrapartum care guideline).^4^ **Table S3**. Categorisation of potential confounders. Table S4. Risk factors known before the onset of labour by maternal BMI category, ‘all risks’ sample. **Table S5**. Admission to a neonatal unit or intrapartum stillbirth/early neonatal death (perinatal composite outcome) by maternal BMI category, healthy women without additional risk factors and ‘all risks’ sample. **Table S6**. Admission to a neonatal unit or intrapartum stillbirth/early neonatal death (perinatal composite outcome) by maternal BMI category and parity, healthy women without additional risk factors and ‘all risks’ sample. **Table S7**. Individual obstetric interventions and adverse maternal outcomes by maternal BMI category, healthy women without additional risk factors. **Table S8**. Obstetric interventions and adverse maternal outcomes (composite maternal outcome) by BMI category, healthy women without additional risk factors. **Table S9**. Neonatal unit admission or intrapartum stillbirth/ early neonatal death (composite perinatal outcome) by maternal BMI category and planned birth setting, healthy women without additional risk factors. **Table S10**. Obstetric interventions and adverse maternal outcomes (composite maternal outcome) by maternal BMI category and planned birth setting, healthy women without additional risk factors. **Table S11**. Individual obstetric interventions and adverse maternal outcomes by maternal BMI category, ‘all risks’ sample. **Table S12**. Obstetric interventions and adverse maternal outcomes (composite measure) by maternal BMI category and parity, ‘all risks’ sample.
