# Supplementary material for: The impact of maternal obesity on intrapartum outcomes in otherwise low risk women: secondary analysis of the Birthplace national prospective cohort study
Source: BJOG. 2013 Sep 11;121(3):343–55. doi: 10.1111/1471-0528.12437 (PMC3906828; doi:10.1111/1471-0528.12437)
Supplement: Supplementary file 1 — Table S1. Medical ‘risk factors’ (from NICE intrapartum care guideline).4 Table S2. Obstetric history ‘risk factors’ (from NICE intrapartum care guideline).4 Table S3. Categorisation of potential confounders. Table S4. Risk factors known before the onset of labour by maternal BMI category, ‘all risks’ sample. Table S5. Admission to a neonatal unit or intrapartum stillbirth/early neonatal death (perinatal composite outcome) by maternal BMI category, healthy women without additional risk factors and ‘all risks’ sample. Table S6. Admission to a neonatal unit or intrapartum stillbirth/early neonatal death (perinatal composite outcome) by maternal BMI category and parity, healthy women without additional risk factors and ‘all risks’ sample. Table S7. Individual obstetric interventions and adverse maternal outcomes by maternal BMI category, healthy women without additional risk factors. Table S8. Obstetric interventions and adverse maternal outcomes (composite maternal outcome) by BMI category, healthy women without additional risk factors. Table S9. Neonatal unit admission or intrapartum stillbirth/ early neonatal death (composite perinatal outcome) by maternal BMI category and planned birth setting, healthy women without additional risk factors. Table S10. Obstetric interventions and adverse maternal outcomes (composite maternal outcome) by maternal BMI category and planned birth setting, healthy women without additional risk factors. Table S11. Individual obstetric interventions and adverse maternal outcomes by maternal BMI category, ‘all risks’ sample. Table S12. Obstetric interventions and adverse maternal outcomes (composite measure) by maternal BMI category and parity, ‘all risks’ sample. [file bjo0121-0343-sd1.pdf]

Supplementary tables S1 to S12

Table S1: Medical 'risk factors' (from NICE intrapartum care guideline)

Table S2: Obstetric history 'risk factors' (from NICE intrapartum care guideline)

Table S3: Categorisation of potential confounders

Table S4. Risk factors known prior to the onset of labour by maternal BMI category, 'all risks' sample

Table S5: Admission to a neonatal unit or intrapartum stillbirth/early neonatal death (perinatal composite outcome) by maternal BMI category, healthy women without additional risk factors and 'all risks' sample

Table S6: Admission to a neonatal unit or intrapartum stillbirth/early neonatal death (perinatal composite outcome) by maternal BMI category and parity, healthy women without additional risk factors and 'all risks' sample

Table S7: Individual obstetric interventions and adverse maternal outcomes by maternal BMI category, healthy women without additional risk factors

Table S8: Obstetric interventions and adverse maternal outcomes (composite maternal outcome) by BMI category, healthy women without additional risk factors

Table S9: Neonatal unit admission or intrapartum stillbirth/early neonatal death (composite perinatal outcome) by maternal BMI category and planned birth setting, healthy women without additional risk factors

Table S10: Obstetric interventions and adverse maternal outcomes (composite maternal outcome) by maternal BMI category and planned birth setting, healthy women without additional risk factors

Table S11: Individual obstetric interventions and adverse maternal outcomes by maternal BMI category, 'all risks' sample

Table S12: Obstetric interventions and adverse maternal outcomes (composite measure) by maternal BMI category and parity, 'all risks' sample

Table S1: Medical 'risk factors' (from NICE intrapartum care guideline)

System	Condition
Cardiovascular	Confirmed cardiac disease
	Hypertensive disorders
Respiratory	Asthma requiring an increase in treatment or hospital treatment
	Cystic fibrosis
Haematological	Haemoglobinopathies – sickle-cell disease, beta-thalassaemia major
	History of thromboembolic disorders
	Immune thrombocytopenia purpura or other platelet disorder or platelet count below 100 000
	Von Willebrand's disease
	Bleeding disorder in the woman or unborn baby
	Atypical antibodies which carry a risk of haemolytic disease of the newborn
Infective	Risk factors associated with group B streptococcus whereby antibiotics in labour would be recommended
	Hepatitis B/C with abnormal liver function tests
	Infected with HIV
	Toxoplasmosis – women receiving treatment
	Current active infection of chicken pox/rubella/genital herpes in the woman or baby
	Tuberculosis under treatment
Immune	Systemic lupus erythematosus
	Scleroderma
Endocrine	Hyperthyroidism
	Diabetes
Renal	Abnormal renal function
	Renal disease requiring supervision by a renal specialist
Neurological	Epilepsy
	Myasthenia gravis
	Previous cerebrovascular accident
Gastrointestinal	Liver disease associated with current abnormal liver function tests
Psychiatric	Psychiatric disorder requiring current inpatient care

Table S2: Obstetric history 'risk factors' (from NICE intrapartum care guideline)

**Type of condition**	**Condition or event**
Previous complications	Unexplained stillbirth/neonatal death or previous death related to intrapartum difficulty
	Previous baby with neonatal encephalopathy
	Pre-eclampsia requiring preterm birth
	Placental abruption with adverse outcome
	Eclampsia
	Uterine rupture
	Primary postpartum haemorrhage requiring additional treatment or blood transfusion
	Retained placenta requiring manual removal in theatre
	Caesarean section
	Shoulder dystocia
Current pregnancy	Multiple birth
	Placenta praevia
	Pre-eclampsia or pregnancy-induced hypertension
	Preterm labour or preterm pre-labour rupture of membranes
	Placental abruption
	Anaemia – haemoglobin less than 8.5 g/dl at onset of labour
	Confirmed intrauterine death
	Induction of labour
	Substance misuse
	Alcohol dependency requiring assessment or treatment
	Onset of gestational diabetes
	Malpresentation – breech or transverse lie
	Body mass index at booking of greater than 35 kg/m^2
	Recurrent antepartum haemorrhage
	Small for gestational age in this pregnancy (less than fifth centile or reduced growth velocity on ultrasound)
Fetal indication	Abnormal fetal heart rate (FHR)/Doppler studies
	Ultrasound diagnosis of oligo-/polyhydramnios
Gynaecological history	Myomectomy
	Hysterotomy

Table S3: Categorisation of potential confounders

Covariate	Response categories
Maternal age	1 Less than 20 years<br>2 20 to 24 years<br>3 25 to 29 years<br>4 30 to 34 years<br>5 35 to 39 years<br>6 40+ years
Ethic group	1 White<br>2 Indian or Bangladeshi<br>3 Pakistani<br>4 Black Caribbean<br>5 Black African<br>6 Mixed<br>7 Other
Understanding of English	1 Fluent<br>2 Some understanding/able to communicate verbally<br>3 No understanding/not able to communicate verbally
Marital or partner status	1 Married/living with partner<br>2 Single/unsupported by partner
Index of Multiple Deprivation score	1 1st quintile (least deprived)<br>2 2nd quintile<br>3 3rd quintile<br>4 4th quintile<br>5 5th quintile (most deprived)
Parity<br>(Previous pregnancies ≥24 weeks)	1 Nulliparous<br>2 1 previous<br>3 2 previous<br>4 3 or more previous
Gestation at delivery	1 37 weeks<br>2 38 weeks<br>3 39 weeks<br>4 40 weeks<br>5 41 weeks<br>6 42 to 44 weeks

Table S4. Risk factors known prior to the onset of labour by maternal BMI category, 'all risks' sample

Condition	Underweight n=821		Normal weight n=13143		Overweight n=7560		Obese n=3479		Very obese n=1901	
	n	%	n	%	n	%	n	%	n	%
**Cardiovascular**										
Confirmed cardiac disease	7	0.85	62	0.47	27	0.36	12	0.34	8	0.42
Hypertensive disorders	6	0.73	127	0.97	139	1.84	88	2.53	80	4.21
**Respiratory**										
Asthma*	1	0.12	52	0.40	32	0.42	17	0.49	20	1.05
Cystic Fibrosis	0	-	1	0.01	0	-	3	0.09	0	-
**Haematological**										
Haemoglobinopathies*	1	0.12	13	0.10	11	0.15	5	0.14	1	0.05
Thromboembolic disorders	3	0.37	58	0.44	35	0.46	22	0.63	14	0.74
Thrombocytopenia*	4	0.49	33	0.25	18	0.24	10	0.29	3	0.16
Von Willebrand's disease	0	-	9	0.07	2	0.03	2	0.06	1	0.05
Bleeding disorder	0	-	3	0.02	5	0.07	0	-	1	0.05
Atypical antibodies*	2	0.24	31	0.24	20	0.26	9	0.26	2	0.11
**Infective**										
Group B strep*	29	3.53	441	3.36	270	3.57	126	3.62	75	3.95
Hepatitis B/C/w abnormal LFT	3	0.37	32	0.24	26	0.34	6	0.17	3	0.16
HIV	0	-	6	0.05	2	0.03	4	0.11	0	-
Toxoplasmosis *	0	-	0	-	0	-	0	-	0	-
Current chicken pox/rubella/genital herpes	1	0.12	9	0.07	10	0.13	1	0.03	1	0.05
Tuberculosis under treatment	0	-	0	-	0	-	1	0.03	0	-
**Immune**										
SLE	0	-	10	0.08	6	0.08	1	0.03	1	0.05
Scleroderma	0	-	1	0.01	0	-	0	-	0	-
**Endocrine**										
Hyperthyroidism	2	0.24	53	0.40	45	0.60	23	0.66	2	0.11
Diabetes	1	0.12	79	0.60	52	0.69	68	1.95	52	2.74
**Renal**										
Abnormal renal function	1	0.12	15	0.11	7	0.09	6	0.17	1	0.05
Renal disease*	0	-	12	0.09	9	0.12	5	0.14	2	0.11
**Neurological**										
Epilepsy	2	0.24	72	0.55	54	0.71	22	0.63	14	0.74
Myasthenia gravis	0	-	1	0.01	1	0.01	0	-	0	-
Previous cerebrovascular accident	0	-	3	0.02	3	0.04	3	0.09	0	-
**Gastrointestinal**										
Liver disease*	0	-	24	0.18	12	0.16	5	0.14	4	0.21
**Psychiatric**										
Current inpatient care	0	-	12	0.09	11	0.15	5	0.14	1	0.05
**Other**										
Other medical	7	0.85	94	0.72	41	0.54	21	0.60	10	0.53
**Any medical risk factor**	67	8.16	1187	9.03	783	10.36	433	12.45	265	13.94

* The description of some risk factors has been shortened. See NICE intrapartum care guideline for fuller description of marked risk factors.

Table S4 (continued). Risk factors known prior to the onset of labour by maternal BMI category, 'all risks' sample

Condition	Underweight n=821		Normal weight n=13143		Overweight n=7560		Obese n=3479		Very obese n=1901	
	n	%	n	%	n	%	n	%	n	%
**Previous complications**										
Unexplained stillbirth/neonatal death*	3	0.37	75	0.57	54	0.71	28	0.80	11	0.58
Neonatal encephalopathy	0	-	2	0.02	2	0.03	0	-	0	-
Pre-eclampsia requiring preterm birth	2	0.24	29	0.22	20	0.26	22	0.63	15	0.79
Placental abruption*	0	-	4	0.03	7	0.09	2	0.06	1	0.05
Eclampsia	1	0.12	9	0.07	4	0.05	6	0.17	2	0.11
Uterine rupture	0	-	2	0.02	0	-	0	-	0	-
PPH with treatment/ transfusion	7	0.85	101	0.77	58	0.77	44	1.26	18	0.95
Retained placenta*	5	0.61	85	0.65	27	0.36	24	0.69	12	0.63
Caesarean section	29	3.53	514	3.91	394	5.21	227	6.52	108	5.68
Shoulder dystocia	2	0.24	24	0.18	21	0.28	15	0.43	9	0.47
**Current pregnancy**										
Placenta praevia	0	-	7	0.05	1	0.01	2	0.06	1	0.05
Pre-eclampsia or pregnancy induced hypertension	16	1.95	315	2.40	299	3.96	187	5.38	130	6.84
Preterm labour or preterm PROM	2	0.24	39	0.30	25	0.33	5	0.14	5	0.26
Placental abruption	0	-	10	0.08	6	0.08	1	0.03	1	0.05
Anaemia*	4	0.49	26	0.20	8	0.11	2	0.06	0	-
Induction of labour	93	11.33	2082	15.84	1428	18.89	813	23.37	516	27.14
Substance misuse	9	1.10	77	0.59	35	0.46	6	0.17	4	0.21
Alcohol dependency *	1	0.12	11	0.08	4	0.05	2	0.06	1	0.05
Gestational diabetes	5	0.61	125	0.95	121	1.60	111	3.19	114	6.00
Malpresentation	3	0.37	26	0.20	27	0.36	6	0.17	5	0.26
Recurrent antepartum haemorrhage	6	0.73	28	0.21	7	0.09	9	0.26	5	0.26
**Fetal**										
Small for gestational age*	51	6.21	263	2.00	98	1.30	24	0.69	15	0.79
Abnormal fetal heart rate*	1	0.12	25	0.19	15	0.20	5	0.14	5	0.26
Oligo/polyhydramnios*	13	1.58	116	0.88	82	1.08	45	1.29	35	1.84
**Previous gynaecological history**										
Myomectomy	0	-	2	0.02	3	0.04	0	-	1	0.05
Hysterotomy	0	-	0	-	0	-	0	-	0	-
**Other obstetric**										
Other obstetric	17	2.07	191	1.45	130	1.72	57	1.64	32	1.68
**Any obstetric/fetal risk factor**	198	24.12	3399	25.86	2291	30.30	1264	36.33	779	40.98
**ANY risk factor**	244	29.72	4207	32.01	2782	36.80	1524	34.81	919	48.24
* The description of some risk factors has been shortened. See NICE intrapartum care guideline for fuller description of marked risk factors.										

Table S5: Admission to a neonatal unit or intrapartum stillbirth/early neonatal death (perinatal composite outcome) by maternal BMI category, healthy women without additional risk factors and 'all risks' sample

	Events	Births	Weighted %[1]		Unadjusted[1]		Unadjusted[1,2]		Adjusted[1,3]	
	n	n	%	(95% CI)	RR	(95% CI)	RR	(95% CI)	RR	(95% CI)
**Otherwise healthy women without additional risk factors**										
Underweight	14	576	2.4	(1.4-3.4)	0.84	(0.50-1.39)	0.82	(0.49-1.40)	0.82	(0.48-1.39)
Normal Weight	249	8881	2.8	(2.2-3.5)	1	-	1	-	1	-
Overweight	123	4750	2.7	(2.0-3.5)	0.97	(0.76-1.23)	0.94	(0.73-1.20)	0.96	(0.75-1.23)
Obese	58	1946	3.0	(2.1-4.0)	1.06	(0.76-1.47)	1.11	(0.80-1.53)	1.17	(0.85-1.62)
Very obese	43	981	4.7	(3.2-6.2)	1.64	(1.22-2.21)	1.70	(1.28-2.27)	1.90	(1.41-2.56)
Total	487	17134	2.9	(2.4-3.5)						
**'All risks' sample**										
Underweight	27	818	3.1	(2.1-4.1)	0.88	(0.61-1.25)	0.84	(0.57-1.22)	0.79	(0.54-1.16)
Normal Weight	446	13074	3.5	(2.8-4.2)	1	-	1	-	1	-
Overweight	241	7521	3.4	(2.8-4.1)	0.98	(0.80-1.20)	0.97	(0.79-1.20)	1.01	(0.81-1.26)
Obese	134	3463	4.0	(3.1-5.0)	1.15	(0.95-1.39)	1.14	(0.94-1.39)	1.24	(1.01-1.52)
Very obese	100	1897	5.6	(4.3-6.9)	1.60	(1.34-1.90)	1.64	(1.38-1.94)	1.75	(1.49-2.05)
Total	948	26773	3.7	(3.1-4.3)						

[1] Weighted to reflect each unit's duration of participation and the sampling of OUs.
[2] Restricted to women included in the adjusted analysis.
[3] Adjusted for maternal age, ethnic group, understanding of English, marital/partner status, index of multiple deprivation score quintile, previous pregnancies >=24 weeks, and gestation (completed weeks).

Table S6: Admission to a neonatal unit or intrapartum stillbirth/early neonatal death (perinatal composite outcome) by maternal BMI category and parity, healthy women without additional risk factors and 'all risks' sample

	Events	Births	Weighted[1]		Unadjusted[1]		Unadjusted[1,2]		Adjusted[1,3]	
	n	n	%	(95% CI)	RR	(95% CI)	RR	(95% CI)	RR	(95% CI)
**Healthy women without additional risk factors**										
**Nulliparous women**										
Under`weight	9	344	2.6	(0.9-4.3)	0.70	(0.35-1.40)	0.72	(0.36-1.44)	0.72	(0.36-1.46)
Normal Weight	180	4979	3.7	(2.8-4.6)	1	1	1	1	1	1
Overweight	76	2406	3.3	(2.1-4.6)	0.90	(0.62-1.30)	0.88	(0.61-1.26)	0.88	(0.62-1.24)
Obese	39	938	4.1	(2.5-5.8)	1.11	(0.75-1.65)	1.16	(0.79-1.72)	1.18	(0.80-1.74)
Very obese	28	417	7.1	(4.3-9.9)	1.90	(1.24-2.91)	1.96	(1.28-2.99)	2.00	(1.31-3.05)
Total	332	9084	3.8	(3.0-4.6)						
**Multiparous women**										
Underweight	5	232	2.0	(0.4-3.7)	1.20	(0.48-2.98)	1.05	(0.38-2.92)	1.13	(0.40-3.19)
Normal Weight	68	3891	1.7	(1.2-2.2)	1	1	1	1	1	1
Overweight	46	2333	2.1	(1.5-2.7)	1.23	(0.92-1.63)	1.17	(0.87-1.58)	1.19	(0.88-1.61)
Obese	19	1005	2.0	(1.0-2.9)	1.16	(0.65-2.07)	1.18	(0.66-2.10)	1.26	(0.69-2.28)
Very obese	15	563	2.9	(1.5-4.3)	1.69	(1.13-2.53)	1.72	(1.15-2.57)	1.83	(1.22-2.75)
Total	153	8024	1.9	(1.5-2.4)						
**'All risks' sample**										
**Nulliparous women**										
Underweight	19	480	3.8	(2.1-5.5)	0.87	(0.54-1.39)	0.83	(0.50-1.38)	0.80	(0.47-1.36)
Normal Weight	298	7048	4.4	(3.4-5.3)	1	-	1	-	1	-
Overweight	129	3634	3.9	(2.7-5.1)	0.90	(0.66-1.22)	0.89	(0.66-1.21)	0.90	(0.67-1.21)
Obese	77	1595	5.0	(3.6-6.3)	1.14	(0.91-1.44)	1.16	(0.91-1.46)	1.20	(0.95-1.52)
Very obese	59	810	7.7	(5.7-9.6)	1.76	(1.36-2.27)	1.79	(1.38-2.31)	1.81	(1.40-2.34)
Total	582	13567	4.5	(3.6-5.3)						
**Multiparous women**										
Underweight	8	337	2.1	(0.8-3.4)	0.84	(0.41-1.71)	0.78	(0.38-1.62)	0.72	(0.36-1.46)
Normal Weight	147	6012	2.5	(2.0-3.0)	1	-	1	-	1	-
Overweight	111	3872	3.0	(2.5-3.5)	1.20	(0.96-1.51)	1.18	(0.93-1.50)	1.21	(0.94-1.56)
Obese	57	1863	3.2	(2.2-4.2)	1.30	(0.94-1.79)	1.25	(0.90-1.74)	1.32	(0.95-1.84)
Very obese	41	1086	4.1	(2.6-5.6)	1.63	(1.21-2.20)	1.66	(1.24-2.23)	1.66	(1.23-2.24)
Total	364	13170	2.9	(2.4-3.3)						

[1] Weighted to reflect each unit's duration of participation and the sampling of OUs.
[2] Restricted to women included in the adjusted analysis.
[3] Adjusted for maternal age, ethnic group, understanding of English, marital/partner status, index of multiple deprivation score quintile, previous pregnancies >=24 weeks, and gestation (completed weeks).

Table S7: Individual obstetric interventions and adverse maternal outcomes by maternal BMI category, healthy women without additional risk factors

	Events	Births	Weighted %[1]		Unadjusted[1]		Unadjusted[1,2]		Adjusted[1,3]	
	n	n	%	(95% CI)	RR	(95% CI)	RR	(95% CI)	RR	(95% CI)
**Instrumental delivery**										
Underweight	79	577	14.0	(11.0-17.0)	0.86	(0.72-1.02)	0.88	(0.74-1.04)	0.95	(0.79-1.13)
Normal Weight	1397	8928	16.3	(13.9-18.7)	1	-	1	-	1	-
Overweight	635	4774	13.5	(11.8-15.2)	0.83	(0.76-0.91)	0.83	(0.76-0.91)	0.87	(0.80-0.95)
Obese	249	1951	12.8	(10.8-14.9)	0.79	(0.67-0.93)	0.80	(0.67-0.95)	0.86	(0.74-1.00)
Very obese	84	983	9.4	(7.0-11.7)	0.57	(0.46-0.71)	0.59	(0.48-0.73)	0.70	(0.57-0.86)
Total	2444	17213	14.7	(12.7-16.6)						
**Intrapartum caesarean section**										
Underweight	39	577	6.9	(4.5-9.4)	0.72	(0.54-0.97)	0.75	(0.56-1.00)	0.83	(0.61-1.13)
Normal Weight	846	8928	9.6	(8.3-10.9)	1	-	1	-	1	-
Overweight	588	4774	12.5	(11.1-13.8)	1.30	(1.16-1.46)	1.31	(1.17-1.46)	1.34	(1.20-1.50)
Obese	260	1951	13.6	(11.7-15.6)	1.42	(1.24-1.64)	1.41	(1.22-1.64)	1.52	(1.30-1.79)
Very obese	135	983	13.6	(11.2-16.0)	1.42	(1.16-1.74)	1.47	(1.19-1.80)	1.69	(1.35-2.12)
Total	1868	17213	11.0	(9.8-12.1)						
**Augmentation**										
Underweight	102	571	17.4	(13.7-21.1)	0.75	(0.61-0.93)	0.77	(0.61-0.96)	0.80	(0.66-0.98)
Normal Weight	2024	8823	23.2	(21.2-25.1)	1	-	1	-	1	-
Overweight	1152	4753	24.6	(22.1-27.1)	1.06	(1.00-1.12)	1.06	(0.99-1.12)	1.10	(1.03-1.16)
Obese	519	1930	27.3	(23.7-30.9)	1.18	(1.08-1.29)	1.19	(1.08-1.30)	1.26	(1.16-1.37)
Very obese	266	972	27.2	(22.9-31.5)	1.18	(1.03-1.35)	1.19	(1.05-1.36)	1.35	(1.20-1.53)
Total	4063	17031	24.1	(21.9-26.2)						
**General anaesthesia**										
Underweight	8	559	1.4	(0.3-2.6)	1.09	(0.51-2.34)	1.12	(0.52-2.39)	1.16	(0.53-2.54)
Normal Weight	117	8805	1.3	(1.0-1.6)	1	-	1	-	1	-
Overweight	80	4707	1.7	(1.2-2.1)	1.25	(0.94-1.68)	1.24	(0.92-1.66)	1.25	(0.93-1.69)
Obese	31	1920	1.6	(1.0-2.2)	1.19	(0.79-1.81)	1.15	(0.75-1.77)	1.19	(0.78-1.81)
Very obese	20	971	2.1	(1.0-3.2)	1.62	(0.96-2.73)	1.67	(1.00-2.81)	1.79	(1.04-3.07)
Total	256	16972	1.5	(1.2-1.8)						

[1] Weighted to reflect each unit’s duration of participation and the sampling of OUs.
[2] Restricted to women included in the adjusted analysis.
[3] Adjusted for maternal age, ethnic group, understanding of English, marital/partner status, index of multiple deprivation score quintile, previous pregnancies >=24 weeks, and gestation (completed weeks).

Table S7 (continued): Individual obstetric interventions and adverse maternal outcomes by maternal BMI category, healthy women without additional risk factors

	Events	Births	Weighted %[1]		Unadjusted[1]		Unadjusted[1,2]		Adjusted[1,3]	
	n	n	%	(95% CI)	RR	(95% CI)	RR	(95% CI)	RR	(95% CI)
**Maternal blood transfusion**										
Underweight	6	574	1.2	(0.4-2.1)	0.94	(0.44-2.02)	0.99	(0.46-2.14)	1.03	(0.48-2.21)
Normal Weight	112	8881	1.3	(1.0-1.7)	1	-	1	-	1	-
Overweight	61	4735	1.2	(0.9-1.6)	0.91	(0.61-1.35)	0.92	(0.61-1.40)	0.96	(0.62-1.48)
Obese	25	1945	1.2	(0.7-1.8)	0.94	(0.63-1.40)	0.94	(0.61-1.45)	1.00	(0.65-1.53)
Very obese	9	984	0.9	(0.4-1.4)	0.66	(0.34-1.28)	0.70	(0.36-1.35)	0.77	(0.40-1.50)
Total	213	17119	1.3	(1.0-1.5)						
**3rd / 4th degree tear**										
Underweight	20	571	3.6	(2.3-5.0)	1.10	(0.75-1.60)	1.08	(0.73-1.59)	1.15	(0.78-1.68)
Normal Weight	301	8903	3.3	(2.8-3.9)	1	-	1	-	1	-
Overweight	139	4759	2.9	(2.4-3.5)	0.88	(0.74-1.04)	0.88	(0.74-1.05)	0.93	(0.78-1.10)
Obese	48	1947	2.4	(1.7-3.1)	0.73	(0.53-1.02)	0.74	(0.53-1.03)	0.82	(0.60-1.13)
Very obese	24	983	2.4	(1.3-3.7)	0.75	(0.47-1.20)	0.71	(0.45-1.12)	0.86	(0.56-1.33)
Total	532	17163	3.1	(2.6-3.5)						
**Maternal admission for higher level care**										
Underweight	5	577	1.0	(0.1-1.9)	1.46	(0.60-3.58)	1.47	(0.60-3.60)	1.63	(0.72-3.69)
Normal Weight	57	8936	0.7	(0.3-1.0)	1	-	1	-	1	-
Overweight	28	4778	0.6	(0.2-0.9)	0.80	(0.42-1.50)	0.78	(0.40-1.50)	0.78	(0.41-1.49)
Obese	11	1955	0.7	(0.2-1.1)	0.96	(0.56-1.64)	0.87	(0.48-1.57)	0.88	(0.50-1.54)
Very obese	5	984	0.5	(0.1-0.9)	0.68	(0.26-1.74)	0.69	(0.27-1.77)	0.71	(0.25-2.03)
Total	106	17230	0.6	(0.4-0.9)						

[1] Weighted to reflect each unit's duration of participation and the sampling of OUs.
[2] Restricted to women included in the adjusted analysis.
[3] Adjusted for maternal age, ethnic group, understanding of English, marital/partner status, index of multiple deprivation score quintile, previous pregnancies >=24 weeks, and gestation (completed weeks).

Table S8: Obstetric interventions and adverse maternal outcomes (composite maternal outcome) by BMI category, healthy women without additional risk factors

	Events	Births	Weighted %[1]		Unadjusted[1]		Unadjusted[1,2]		Adjusted[1,3]	
	n	n	%	(95% CI)	RR	(95% CI)	RR	(95% CI)	RR	(95% CI)
**All healthy women without additional risk factors**										
Underweight	182	558	32.8	(28.9-36.7)	0.88	(0.78-0.99)	0.89	(0.79-1.00)	0.94	(0.84-1.04)
Normal Weight	3192	8648	37.5	(35.1-39.8)	1	-	1	-	1	-
Overweight	1747	4621	38.2	(35.8-40.6)	1.02	(0.97-1.07)	1.02	(0.97-1.07)	1.06	(1.01-1.11)
Obese	748	1885	40.1	(36.7-43.4)	1.07	(0.99-1.15)	1.07	(1.00-1.15)	1.14	(1.08-1.20)
Very obese	342	960	36.4	(31.9-40.8)	0.97	(0.87-1.08)	0.98	(0.88-1.10)	1.12	(1.02-1.23)
Total	6211	16672	37.7	(35.6-39.9)						
**Healthy women by parity**										
**Nulliparous women**										
Underweight	150	330	45.6	(39.5-51.7)	0.86	(0.76-0.98)	0.87	(0.76-0.99)	0.94	(0.82-1.09)
Normal Weight	2524	4833	52.9	(50.3-55.4)	1	-	1	-	1	-
Overweight	1277	2321	55.7	(52.4-59.0)	1.05	(1.01-1.10)	1.05	(1.00-1.09)	1.04	(0.99-1.08)
Obese	535	907	60.2	(55.9-64.4)	1.14	(1.07-1.21)	1.13	(1.07-1.20)	1.12	(1.05-1.18)
Very obese	225	404	57.1	(52.2-62.0)	1.08	(0.98-1.19)	1.09	(0.99-1.19)	1.08	(0.99-1.18)
Total	4711	8795	54.3	(51.8-56.8)						
**Multiparous women**										
Underweight	32	228	14.6	(8.1-21.1)	0.83	(0.55-1.24)	0.86	(0.57-1.28)	0.87	(0.57-1.31)
Normal Weight	666	3809	17.7	(15.7-19.7)	1	-	1	-	1	-
Overweight	465	2290	20.2	(17.7-22.7)	1.14	(1.00-1.30)	1.16	(1.02-1.32)	1.16	(1.02-1.32)
Obese	212	975	21.3	(17.6-25.0)	1.20	(1.01-1.44)	1.19	(1.01-1.41)	1.22	(1.05-1.42)
Very obese	117	555	21.0	(15.1-26.9)	1.19	(0.90-1.57)	1.19	(0.90-1.58)	1.24	(0.97-1.59)
Total	1492	7857	19.0	(17.1-21.0)						

[1] Weighted to reflect each unit's duration of participation and the sampling of OUs.
[2] Restricted to women included in the adjusted analysis.
[3] Adjusted for maternal age, ethnic group, understanding of English, marital/partner status, index of multiple deprivation score quintile, previous pregnancies >=24 weeks, and gestation (completed weeks).

Table S9: Neonatal unit admission or intrapartum stillbirth/early neonatal death (composite perinatal outcome) by maternal BMI category and planned birth setting, healthy women without additional risk factors

	Events	Births	Weighted %[1]		Unadjusted[1]		Unadjusted[1,2]		Adjusted[1,3]	
	n	n	%	(95% CI)	RR	(95% CI)	RR	(95% CI)	RR	(95% CI)
**OU**										
Underweight	14	576	2.4	(1.4-3.4)	0.84	(0.50-1.39)	0.82	(0.49-1.40)	0.81	(0.48-1.39)
Normal Weight	249	8881	2.8	(2.2-3.5)	1	-	1	-	1	-
Overweight	123	4750	2.7	(2.0-3.5)	0.97	(0.76-1.23)	0.94	(0.73-1.20)	0.96	(0.75-1.24)
Obese	58	1946	3.0	(2.1-4.0)	1.06	(0.76-1.47)	1.11	(0.80-1.53)	1.18	(0.85-1.63)
Very obese*	34	672	5.4	(3.6-7.2)	1.89	(1.37-2.60)	1.95	(1.42-2.67)	2.16	(1.57-2.98)
Total	478	16825	2.9	(2.4-3.5)						
**Home**										
Underweight	7	321	1.8	(0.3-3.2)	1.02	(0.44-2.36)	1.02	(0.44-2.38)	1.11	(0.47-2.63)
Normal Weight	135	8088	1.7	(1.4-2.1)	1	-	1	-	1	-
Overweight	70	3750	1.8	(1.4-2.3)	1.05	(0.77-1.42)	1.06	(0.78-1.44)	1.09	(0.81-1.47)
Obese	22	1224	2.1	(1.0-3.1)	1.20	(0.70-2.05)	1.22	(0.71-2.10)	1.36	(0.80-2.29)
Very obese*	5	263	1.7	(0.2-3.2)	0.99	(0.41-2.42)	1.01	(0.42-2.46)	1.17	(0.49-2.81)
Total	239	13646	1.8	(1.5-2.1)						
**FMU**										
Underweight	5	236	2.1	(0.2-3.9)	1.22	(0.45-3.30)	1.26	(0.46-3.40)	1.29	(0.46-3.61)
Normal Weight	95	5623	1.7	(1.0-2.3)	1	1	1	1	1	1
Overweight	50	2673	1.8	(1.2-2.4)	1.07	(0.72-1.59)	1.09	(0.73-1.62)	1.15	(0.78-1.69)
Obese	19	915	2.0	(1.0-3.0)	1.18	(0.69-2.02)	1.21	(0.71-2.07)	1.33	(0.79-2.25)
Very obese*	3	63	5.1	(0.0-11.4)	3.01	(0.79-11.4)	3.02	(0.79-11.4)	3.95	(1.07-14.6)
Total	172	9510	1.8	(1.3-2.3)						
**AMU**										
Underweight	3	440	0.6	(0.0-1.3)	0.33	(0.12-0.90)	0.34	(0.12-0.93)	0.33	(0.13-0.86)
Normal Weight	144	8196	2.0	(1.4-2.5)	1	1	1	1	1	1
Overweight	77	3781	2.0	(1.4-2.7)	1.04	(0.74-1.47)	1.07	(0.75-1.52)	1.15	(0.78-1.68)
Obese	30	1262	2.4	(1.3-3.4)	1.21	(0.73-2.01)	1.18	(0.68-2.05)	1.33	(0.75-2.37)
Very obese*	1	138	0.9	(0.0-2.3)	0.46	(0.97-2.17)	0.47	(0.10-2.22)	0.62	(0.15-2.59)
Total	255	13817	2.0	(1.5-2.4)						

*All analyses in this table were restricted to women with a BMI≤40.
[1] Weighted to reflect each unit's duration of participation and the sampling of OUs.
[2] Restricted to women included in the adjusted analysis.
[3] Adjusted for maternal age, ethnic group, understanding of English, marital/partner status, index of multiple deprivation score quintile, previous pregnancies >=24 weeks, and gestation (completed weeks).

Table S10: Obstetric interventions and adverse maternal outcomes (composite maternal outcome) by maternal BMI category and planned birth setting, healthy women without additional risk factors

	Events	Births	Weighted %[1]		Unadjusted[1]		Unadjusted[1,2]		Adjusted[1,3]	
	n	n	%	(95% CI)	RR	(95% CI)	RR	(95% CI)	RR	(95% CI)
**OU**										
Underweight	182	558	32.8	(28.9-36.7)	0.88	(0.78-0.99)	0.89	(0.79-1.00)	0.94	(0.84-1.05)
Normal Weight	3192	8648	37.5	(35.1-39.8)	1	-	1	-	1	-
Overweight	1747	4621	38.2	(35.8-40.6)	1.02	(0.97-1.07)	1.02	(0.97-1.07)	1.06	(1.01-1.11)
Obese	748	1885	40.1	(36.7-43.4)	1.07	(0.99-1.15)	1.07	(1.00-1.15)	1.14	(1.08-1.20)
Very obese*	234	655	36.4	(31.6-41.1)	0.97	(0.87-1.08)	0.98	(0.87-1.10)	1.10	(1.00-1.22)
Total	6103	16367	37.8	(35.6-39.9)						
**Home**										
Underweight	29	318	9.1	(5.4-12.8)	0.80	(0.55-1.18)	0.80	(0.55-1.17)	0.97	(0.68-1.41)
Normal Weight	901	8051	11.3	(10.2-12.5)	1	-	1	-	1	-
Overweight	396	3723	10.2	(9.0-11.3)	0.90	(0.80-1.01)	0.90	(0.80-1.01)	1.03	(0.93-1.14)
Obese	109	1211	8.9	(7.3-10.6)	0.79	(0.65-0.96)	0.79	(0.65-0.96)	1.04	(0.89-1.22)
Very obese*	19	265	6.2	(3.1-9.3)	0.55	(0.33-0.91)	0.55	(0.33-0.92)	0.95	(0.59-1.52)
Total	1454	13568	10.6	(9.7-11.6)						
**FMU**										
Underweight	25	234	11.5	(6.3-16.8)	0.86	(0.53-1.38)	0.85	(0.52-1.38)	0.98	(0.61-1.57)
Normal Weight	813	5584	13.5	(11.8-15.2)	1	-	1	-	1	-
Overweight	369	2650	13.3	(12.0-14.7)	0.99	(0.88-1.11)	0.96	(0.87-1.07)	1.10	(0.98-1.22)
Obese	86	911	8.7	(6.4-11.0)	0.64	(0.52-0.80)	0.62	(0.51-0.76)	0.74	(0.61-0.89)
Very obese*	4	62	7.0	(0.0-14.3)	0.52	(0.18-1.48)	0.52	(0.18-1.47)	0.80	(0.33-1.94)
Total	1297	9441	12.9	(11.5-14.3)						
**AMU**										
Underweight	88	434	20.1	(13.7-26.6)	0.96	(0.73-1.27)	0.96	(0.73-1.27)	1.08	(0.83-1.42)
Normal Weight	1647	8140	20.9	(19.0-22.8)	1	-	1	-	1	-
Overweight	690	3735	18.6	(16.3-20.9)	0.89	(0.80-0.98)	0.89	(0.81-0.99)	1.02	(0.93-1.13)
Obese	212	1253	16.5	(13.1-19.9)	0.79	(0.67-0.93)	0.79	(0.66-0.93)	1.00	(0.86-1.16)
Very obese*	14	136	11.9	(4.7-19.1)	0.57	(0.32-1.02)	0.58	(0.32-1.05)	0.89	(0.50-1.57)
Total	2651	13698	19.8	(17.8-21.8)						

* All analyses in this table were restricted to women with a BMI≤40.
[1] Weighted to reflect each unit's duration of participation and the sampling of OUs.
[2] Restricted to women included in the adjusted analysis.
[3] Adjusted for maternal age, ethnic group, understanding of English, marital/partner status, index of multiple deprivation score quintile, previous pregnancies >=24 weeks, and gestation (completed weeks).

Table S11: Individual obstetric interventions and adverse maternal outcomes by maternal BMI category, 'all risks' sample

	Events	Births	Weighted[1]		Unadjusted[1]		Unadjusted[1,2]		Adjusted[1,3]	
	n	n	%	(95% CI)	RR	(95% CI)	RR	(95% CI)	RR	(95% CI)
**Instrumental delivery**										
Underweight	118	820	14.9	(12.2-17.6)	0.89	(0.75-1.07)	0.91	(0.76-1.08)	0.96	(0.81-1.13)
Normal Weight	2113	13132	16.7	(14.6-18.8)	1	-	1	-	1	-
Overweight	1019	7554	13.7	(12.1-15.3)	0.82	(0.77-0.88)	0.83	(0.77-0.89)	0.88	(0.83-0.92)
Obese	422	3474	12.3	(10.8-13.8)	0.74	(0.66-0.82)	0.74	(0.66-0.83)	0.81	(0.74-0.89)
Very obese	178	1899	9.8	(8.1-11.5)	0.59	(0.50-0.68)	0.59	(0.51-0.68)	0.68	(0.60-0.78)
Total	3850	26879	14.8	(13.1-16.5)						
**Intrapartum caesarean section**										
Underweight	63	820	7.8	(6.0-9.6)	0.66	(0.55-0.80)	0.66	(0.54-0.81)	0.73	(0.59-0.90)
Normal Weight	1509	13132	11.7	(10.5-13.0)	1	-	1	-	1	-
Overweight	1174	7554	15.7	(14.4-17.0)	1.34	(1.24-1.44)	1.35	(1.26-1.45)	1.37	(1.27-1.47)
Obese	605	3474	17.9	(15.8-19.9)	1.52	(1.35-1.72)	1.53	(1.36-1.72)	1.60	(1.42-1.81)
Very obese	378	1899	20.0	(17.8-22.2)	1.71	(1.48-1.97)	1.73	(1.49-2.00)	1.93	(1.65-2.27)
Total	3729	26879	14.1	(13.0-15.2)						
**Augmentation**										
Underweight	165	811	19.8	(16.7-23.0)	0.70	(0.60-0.81)	0.70	(0.60-0.82)	0.72	(0.62-0.84)
Normal Weight	3644	12977	28.5	(25.7-31.3)	1	-	1	-	1	-
Overweight	2318	7489	31.0	(28.2-33.8)	1.09	(1.04-1.14)	1.09	(1.04-1.14)	1.12	(1.07-1.17)
Obese	1212	3437	35.4	(32.1-38.7)	1.24	(1.17-1.31)	1.24	(1.18-1.31)	1.30	(1.23-1.36)
Very obese	735	1881	39.0	(35.5-42.4)	1.37	(1.26-1.48)	1.38	(1.28-1.49)	1.47	(1.36-1.59)
Total	8074	26595	30.5	(27.8-33.2)						
**General anaesthesia**										
Underweight	15	811	1.9	(0.7-3.0)	1.14	(0.62-2.10)	1.07	(0.55-2.07)	1.05	(0.53-2.05)
Normal Weight	213	12932	1.6	(1.3-2.0)	1	-	1	-	1	-
Overweight	159	7441	2.0	(1.5-2.5)	1.26	(0.97-1.63)	1.23	(0.95-1.62)	1.26	(0.97-1.62)
Obese	80	3414	2.4	(1.7-3.1)	1.46	(1.06-2.00)	1.43	(1.02-2.01)	1.48	(1.08-2.02)
Very obese	55	1871	3.0	(2.3-3.7)	1.85	(1.52-2.25)	1.90	(1.56-2.32)	2.01	(1.67-2.42)
Total	522	26469	1.9	(1.6-2.3)						

[1] Weighted to reflect each unit's duration of participation and the sampling of OUs.
[2] Restricted to women included in the adjusted analysis.
[3] Adjusted for maternal age, ethnic group, understanding of English, marital/partner status, index of multiple deprivation score quintile, previous pregnancies >=24 weeks, and gestation (completed weeks).

Table S11 (continued): Individual obstetric interventions and adverse maternal outcomes by maternal BMI category, 'all risks' sample

	Events	Births	Weighted[1]		Unadjusted[1]		Unadjusted[1,2]		Adjusted[1,3]	
	n	n	%	(95% CI)	RR	(95% CI)	RR	(95% CI)	RR	(95% CI)
**Maternal blood transfusion**										
Underweight	12	816	1.7	(0.5-2.9)	1.11	(0.53-2.34)	1.18	(0.55-2.49)	1.19	(0.57-2.49)
Normal Weight	195	13063	1.6	(1.3-1.9)	1	-	1	-	1	-
Overweight	123	7500	1.6	(1.2-1.9)	1.00	(0.74-1.35)	1.03	(0.77-1.39)	1.06	(0.78-1.42)
Obese	57	3460	1.6	(1.1-2.2)	1.04	(0.77-1.40)	1.08	(0.82-1.42)	1.12	(0.86-1.47)
Very obese	26	1899	1.3	(0.8-1.9)	0.86	(0.52-1.41)	0.91	(0.55-1.49)	0.98	(0.61-1.60)
Total	413	26738	1.6	(1.3-1.8)						
**3rd / 4th degree tear**										
Underweight	25	811	3.3	(2.0-4.5)	1.00	(0.68-1.46)	1.00	(0.69-1.45)	1.01	(0.70-1.47)
Normal Weight	437	13102	3.3	(2.8-3.8)	1	-	1	-	1	-
Overweight	217	7534	2.9	(2.5-3.3)	0.88	(0.76-1.02)	0.90	(0.76-1.05)	0.95	(0.81-1.12)
Obese	88	3468	2.6	(2.0-3.1)	0.78	(0.62-0.97)	0.79	(0.64-0.99)	0.88	(0.70-1.09)
Very obese	42	1895	2.2	(1.4-3.1)	0.68	(0.47-0.97)	0.65	(0.47-0.92)	0.77	(0.55-1.08)
Total	809	26810	3.0	(2.6-3.4)						
**Maternal admission for higher level care**										
Underweight	7	821	0.9	(0.2-1.6)	1.09	(0.53-2.20)	1.11	(0.55-2.25)	1.09	(0.56-2.15)
Normal Weight	106	13143	0.8	(0.5-1.2)	1	-	1	-	1	-
Overweight	56	7560	0.7	(0.4-1.0)	0.83	(0.56-1.24)	0.84	(0.56-1.27)	0.84	(0.56-1.25)
Obese	29	3479	0.9	(0.4-1.5)	1.12	(0.68-1.84)	1.08	(0.67-1.74)	1.08	(0.69-1.70)
Very obese	20	1901	1.0	(0.5-1.5)	1.21	(0.68-2.16)	1.24	(0.69-2.23)	1.32	(0.74-2.35)
Total	218	26904	0.8	(0.6-1.1)						

[1] Weighted to reflect each unit's duration of participation and the sampling of OUs.
[2] Restricted to women included in the adjusted analysis.
[3] Adjusted for maternal age, ethnic group, understanding of English, marital/partner status, index of multiple deprivation score quintile, previous pregnancies >=24 weeks, and gestation (completed weeks).

Table S12: Obstetric interventions and adverse maternal outcomes (composite measure) by maternal BMI category and parity, 'all risks' sample

	Events	Births	Weighted[1]		Unadjusted[1]		Unadjusted[1,2]		Adjusted[1,3]	
	n	n	%	(95% CI)	RR	(95% CI)	RR	(95% CI)	RR	(95% CI)
**'All risks' sample**										
Underweight	287	790	36.6	(33.6-39.5)	0.86	(0.78-0.93)	0.86	(0.79-0.94)	0.89	(0.82-0.97)
Normal Weight	5345	12706	42.7	(40.0-45.5)	1	-	1	-	1	-
Overweight	3302	7315	45.5	(42.9-48.0)	1.06	(1.03-1.10)	1.07	(1.04-1.10)	1.10	(1.07-1.13)
Obese	1623	3357	48.8	(46.0-51.7)	1.14	(1.09-1.19)	1.14	(1.10-1.19)	1.20	(1.15-1.24)
Very obese	906	1848	49.7	(46.2-53.2)	1.16	(1.10-1.24)	1.17	(1.11-1.24)	1.27	(1.20-1.35)
Total	11463	26016	44.6	(42.1-47.1)						
**'All risks', by parity**										
**Nulliparous women**										
Underweight	223	459	48.7	(44.2-53.2)	0.84	(0.77-0.92)	0.84	(0.77-0.92)	0.90	(0.82-0.99)
Normal Weight	3900	6821	58.1	(54.9-61.2)	1	-	1	-	1	-
Overweight	2166	3506	62.4	(59.2-65.5)	1.07	(1.04-1.11)	1.07	(1.04-1.10)	1.06	(1.03-1.09)
Obese	1015	1540	66.8	(63.7-70.0)	1.15	(1.10-1.20)	1.15	(1.10-1.20)	1.13	(1.09-1.18)
Very obese	525	782	67.9	(63.7-72.2)	1.17	(1.09-1.25)	1.18	(1.10-1.25)	1.17	(1.09-1.25)
Total	7829	13108	60.5	(57.6-63.3)						
**Multiparous women**										
Underweight	64	331	20.0	(14.4-25.6)	0.81	(0.62-1.06)	0.82	(0.63-1.08)	0.83	(0.63-1.10)
Normal Weight	1442	5875	24.6	(22.5-26.8)	1	-	1	-	1	-
Overweight	1129	3795	29.8	(27.4-32.1)	1.21	(1.13-1.29)	1.23	(1.14-1.31)	1.21	(1.13-1.30)
Obese	606	1812	33.5	(30.4-36.6)	1.36	(1.25-1.48)	1.35	(1.25-1.46)	1.35	(1.25-1.46)
Very obese	381	1065	36.1	(32.4-39.9)	1.47	(1.32-1.63)	1.47	(1.32-1.63)	1.48	(1.34-1.63)
Total	3622	12878	28.2	(26.2-30.2)						

[1] Weighted to reflect each unit's duration of participation and the sampling of OUs.
[2] Restricted to women included in the adjusted analysis.
[3] Adjusted for maternal age, ethnic group, understanding of English, marital/partner status, index of multiple deprivation score quintile, previous pregnancies >=24 weeks, and gestation (completed weeks).
